# Reduction of *Hox* Gene Expression by Histone H1 Depletion

**DOI:** 10.1371/journal.pone.0038829

**Published:** 2012-06-11

**Authors:** Yunzhe Zhang, Zheng Liu, Magdalena Medrzycki, Kaixiang Cao, Yuhong Fan

**Affiliations:** School of Biology and the Petit Institute for Bioengineering and Bioscience, Georgia Institute of Technology, Atlanta, Georgia, United States of America; National University of Singapore, Singapore

## Abstract

The evolutionarily conserved homeotic (*Hox*) genes are organized in clusters and expressed collinearly to specify body patterning during embryonic development. Chromatin reorganization and decompaction are intimately connected with *Hox* gene activation. Linker histone H1 plays a key role in facilitating folding of higher order chromatin structure. Previous studies have shown that deletion of three somatic H1 subtypes together leads to embryonic lethality and that H1c/H1d/H1e triple knockout (TKO) embryonic stem cells (ESCs) display bulk chromatin decompaction. To investigate the potential role of H1 and higher order chromatin folding in the regulation of *Hox* gene expression, we systematically analyzed the expression of all 39 *Hox* genes in triple H1 null mouse embryos and ESCs by quantitative RT-PCR. Surprisingly, we find that H1 depletion causes significant reduction in the expression of a broad range of *Hox* genes in embryos and ESCs. To examine if any of the three H1 subtypes (H1c, H1d and H1e) is responsible for decreased expression of *Hox* gene in triple-H1 null ESCs, we derived and characterized H1c^−/−^, H1d^−/−^, and H1e^−/−^ single-H1 null ESCs. We show that deletion of individual H1 subtypes results in down-regulation of specific *Hox* genes in ESCs. Finally we demonstrate that, in triple-H1- and single-H1- null ESCs, the levels of H3K4 trimethylation (H3K4me3) and H3K27 trimethylation (H3K27me3) were affected at specific *Hox* genes with decreased expression. Our data demonstrate that marked reduction in total H1 levels causes significant reduction in both expression and the level of active histone mark H3K4me3 at many *Hox* genes and that individual H1 subtypes may also contribute to the regulation of specific *Hox* gene expression. We suggest possible mechanisms for such an unexpected role of histone H1 in *Hox* gene regulation.

## Introduction

The *Hox* genes, encoding a family of evolutionarily conserved transcription factors that contain a DNA binding homeodomain, play fundamental roles in specifying anterior-posterior body patterning during development and are critical for cell fate determination [1]–[3]. The expression levels of *Hox* genes are tightly controlled throughout embryonic development, and aberrant expression and mutation of *Hox* genes can lead to body malformations and multiple types of malignancies [4], [5].

*Hox* genes are organized into genomic clusters and their physical order within the cluster corresponds to their expression order along the anterior-posterior axis. In mammals, there are 39 *Hox* genes arranged in four genomic clusters of thirteen paralog groups (A-D) [6], which are thought to derive from tandem duplication of ancestral genes [7], [8]. Progressive transition of histone modifications and local chromatin decondensation have been found to associate with sequential expression of *Hoxb* and *Hoxd* loci during embryonic development and/or stem cell differentiation [9]–[13]. *Hox* gene clusters are spatially compartmentalized and the transition in their 3D structure corresponds with the changes of H3K4me3 and H3K27me3 [14]. The temporal collinearity of the order of *Hox* gene activation along their physical sequence at genomic loci [15], stepwise transition of chromatin status and spatial configuration [9], [14], and the necessity of the cluster organization for full repression of the entire cluster suggest an important role of chromatin structure in regulation of *Hox* genes [9]-[13]. However, it remains to be determined whether the change of chromatin structure is a contributing factor or a consequence of *Hox* gene activation.

Linker histone H1 is the major chromatin structural protein involved in folding of chromatin into high order structure. H1 binds to the nucleosome and the linker DNA between nucleosomes to promote compaction of nucleosome arrays [16], [17]. Multiple H1 subtypes exist in mammals, providing additional levels of modulation on chromatin structure and function. Among the 11 mammalian H1 subtypes identified, 5 somatic H1 subtypes (H1a-e) are present in abundance in all dividing and non-dividing cells, whereas the replacement H1 (H1^0^) and the 4 germ cell specific H1s are expressed in differentiating cells and germ cells, respectively [18]. Depletion of three somatic H1 subtypes (H1c, H1d, and H1e) together results in embryonic lethality at midgestation, demonstrating the necessity of H1 for mammalian development [19]. We have previously shown that H1c, H1d, and H1e triple knockout (H1 TKO) embryos and embryonic stem cells (ESCs) have marked reduction of total H1 levels and that H1 TKO ESCs display changes in bulk chromatin, including chromatin decondensation, a decreased nucleosome repeat length, as well as reduced levels of histone modifications H3K27me3 and H4K12Ac [19], [20]. Thus H1 TKO embryos and ESCs offer a unique opportunity to examine how the changes in chromatin structure influence *Hox* gene expression.

In the present study, we firstly analyzed the expression changes of all *Hox* genes in H1 TKO embryos and ESCs, and found reduced expression of a distinct set of *Hox* genes in embryos and ESCs, respectively. Furthermore, by characterizing H1c^−/−^; H1d^−/−^; and H1e^−/−^ single-H1 null ESCs established in this study, we showed that individual H1 subtypes regulate specific *Hox* genes in ESCs. Finally we demonstrated that the levels of H3K4me3 were significantly diminished at the affected *Hox* genes in H1 TKO- and single-H1 KO- ESCs, whereas H3K27me3 occupancy was modestly increased at specific *Hox* genes. These results suggest that the marked reduction of H1 levels and decondensation of bulk chromatin cause repression of many *Hox* genes in embryos and ESCs, which may be in part mediated through individual H1 subtypes as well as changes in H3K4me3 and H3K27me3.

## Results

### Loss of H1c, H1d and H1e Leads to Decreased Expression of *Hox* Genes in Embryos and Embryonic Stem Cells

**Figure 1:**
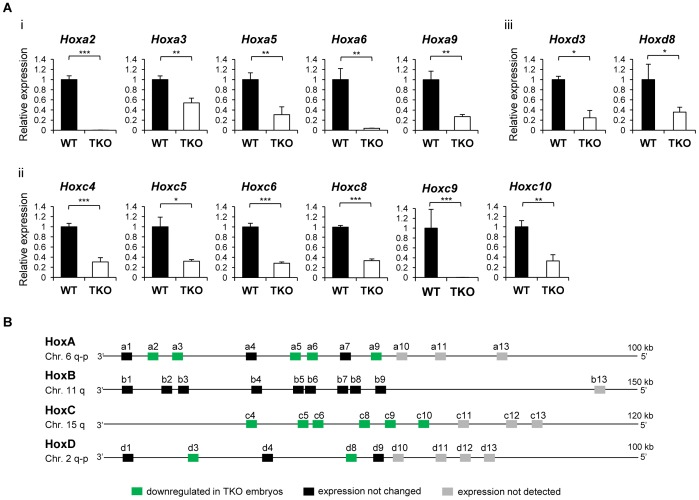
Reduction of *Hox* gene expression in H1 TKO embryos. (A) Relative expression of *Hox* genes with altered mRNA levels in H1 TKO embryos compared with WT. Down-regulated *Hox* genes are located in *HoxA* (i), *HoxC* (ii), and *HoxD* (iii) clusters. Expression levels of *Hox* genes were analyzed by qRT-PCR and normalized over *GAPDH* and represented as a fold change between H1 TKO and WT embryos at E8.5. *: P<0.05, **: P<0.01, ***: P<0.001. Error bars: S.D. (B) The schematic representation of *Hox* gene clusters with expression patterns in H1 TKO embryos compared with WT.

To gain a comprehensive view of the effects histone H1 depletion and changes in bulk chromatin on the regulation of *Hox* gene clusters, we designed a full set of quantitative reverse-transcription PCR assays (qRT-PCR) to measure the expression levels of all 39 murine *Hox* genes across the 4 *Hox* gene clusters in H1 TKO embryos. H1c/H1d/H1e triple heterozygotes were intercrossed to obtain H1 TKO and wild-type (WT) littermate embryos. Most of the H1 TKO embryos display growth retardation and various defects at E9.5 [19]. To minimize the secondary effects caused by broad defects of H1 TKO embryos, we chose to analyze *Hox* gene expression at E8.5 when H1 TKO embryos with comparable size to WT embryos can be recovered. We selected two littermate pairs of WT and H1 TKO embryos at E8.5, and examined the expression patterns of all 39 *Hox* genes using the highly sensitive qRT-PCR assays. As expected, most *Hox* genes were expressed in E8.5 embryos, except the most posterior genes within each cluster (Figure 1). However, surprisingly, many *Hox* genes were expressed at reduced levels in H1 TKO embryos, including *Hoxa2*, *Hoxa3*, *Hoxa5, Hoxa6, Hoxa9*, *Hoxc4*, *Hoxc5*, *Hoxc6*, *Hoxc8*, *Hoxc9*, *Hoxc10, Hoxd3,* and *Hoxd8* (Figure 1). This effect is especially prominent in *Hoxa* and *Hoxc* clusters, in which nearly all of the expressed genes were reduced 3-fold or more (Figure 1A). Interestingly, we did not find increased expression among any of the *Hox* genes (Figure 1B), and none of the *Hoxb* genes were affected in H1 TKO embryos in comparison with WT embryos.

The reduction of expression of many *Hox* genes may cause the growth retardation often observed in H1 TKO embryos at E9.5. However, it remained a formal possibility that the decreased expression of *Hox* genes in H1 TKO embryos was a result of the slight growth retardation presented in the KO embryos, although the H1 TKO embryos used for this analysis were indistinguishable from their WT and heterozygous littermate controls in size and developmental stage. In order to analyze the effects of H1 on a homogeneous cell population, we gauged the effects of H1 depletion on *Hox* gene expression in H1 TKO ESCs. *Hox* genes are repressed by polycomb repressive complexes (PRCs) in ESCs [21]–[26]. Loss of components of either PRC1 or PRC2 in ESCs leads to upregulation of *Hox* genes, presumably due to respective loss of chromatin compaction and H3K27 trimethylase activity [13], [22], [27]. We have shown previously that H1 TKO ESCs have decondensed local chromatin and reduced levels of H3K27m3 in bulk chromatin [20]. We surmise that these changes may lead to elevated levels of expression of specific *Hox* genes. Examination of previous expression data from microarray assays showed that the microarray used for hybridization only contained 11 *Hox* genes, most of which were undetectable in ESCs by the array [20].

**Figure 2:**
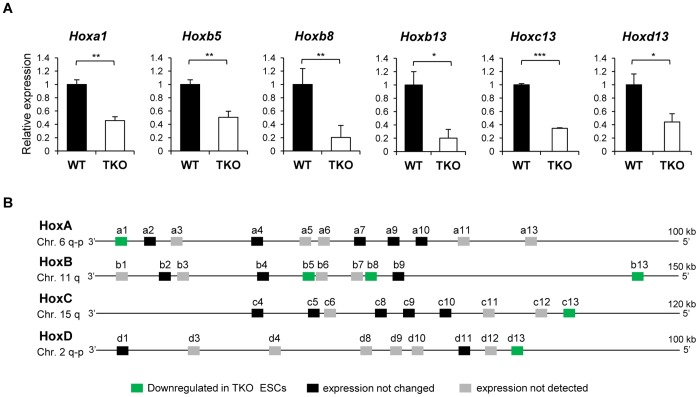
Decreased expression of *Hox* genes in H1 TKO ESCs. (A) Expression analysis of *Hox* genes in WT and H1 TKO ESCs. Y axis and data normalization are as described in the legend to Figure 1. *: P<0.05, **: P<0.01, ***: P<0.001. Error bars: S.D. (B) Expression patterns of *Hox* genes in H1 TKO in comparison with WT ESCs.

We thus applied the qRT-PCR assays to compare the expression levels of all 39 *Hox* genes in WT and TKO ESCs. Consistent with the finding that pluripotent ESCs possess a hyperactive transcriptome [28], we detected expression of 21 *Hox* genes, albeit at low levels, in either or both of WT and H1 TKO ESCs. These genes include *Hoxa1, Hoxa2, Hoxa4, Hoxa7, Hoxa9, Hoxa10, Hoxb2, Hoxb4, Hoxb5, Hoxb8, Hoxb9, Hoxb13, Hoxc4, Hoxc5, Hoxc8, Hoxc9, Hoxc10, Hoxc13, Hoxd1, Hoxd11, and Hoxd13* (Figure 2). Unexpectedly, no increased expression in any of the *Hox* genes was found in H1 TKO ESCs. Instead, the expression levels of 6 *Hox* genes, *Hoxa1, Hoxb5, Hoxb8, Hoxb13, Hoxc13*, and *Hoxd13,* were reduced, with an average of 2–3 fold less in H1 TKO ESCs compared with WT (Figure 2A). Other *Hox* genes did not show consistent changes in expression by loss of H1c, H1d and H1e in ESCs (Figure 2B).

### Specific Regulation of *Hox* Genes in ESCs by Individual H1 Subtypes

To assess the effects of each of the three deleted somatic H1 subtypes in H1 TKO (H1c, H1d and H1e) on *Hox* gene expression in ESCs, we established ESCs that are null for only one of these three H1 subtypes. H1c^−/−^; H1d^−/−^; and H1e^−/−^ mice develop normally and are fertile [29]. Male and female mice homozygous for each single-H1 deletion were bred, H1c^−/−^; H1d^−/−^; and H1e^−/−^ blastocysts were harvested from pregnant female mice at 3.5 day post coitum and their respective single-H1 knockout (KO) ESCs were derived from outgrowth of blastocysts. As shown in metaphase chromosome spreads, the single-H1 KO ESCs had normal karyotypes with 40 chromosomes (Figure S1A) and showed colony morphology typical of undifferentiated ESCs when cultured under conditions promoting self-renewal of ESCs (Figure S1B). They expressed high levels of pluripotency factor OCT4, which is absent in differentiated cells, such as mouse embryonic fibroblasts (MEF) (Figure S1Cii). These single-H1 KO ESCs also had comparable growth rate to WT ESCs (data not shown). Upon differentiation, the single-H1 KO ESCs were able to form embryoid bodies (EB) with characteristic cystic structures and differentiated cell morphologies (Figure S1Ci). As expected, these EBs displayed decreased levels of OCT4 (Figure S1Cii), and increased expression of many differentiation markers, such as *AFP*, *Gata4, T (Brachyury),* and *FLT1*, compared with ESCs (Figure S1Ciii). In addition, teratoma formation analysis indicated that the single-H1 KO ESCs formed typical teratomas containing cells differentiated into all three germ layers after injection into immunodeficient mice (data not shown). These data indicate that any one of these three somatic H1 subtypes is dispensable for self-renewal and differentiation of ESCs.

**Figure 3:**
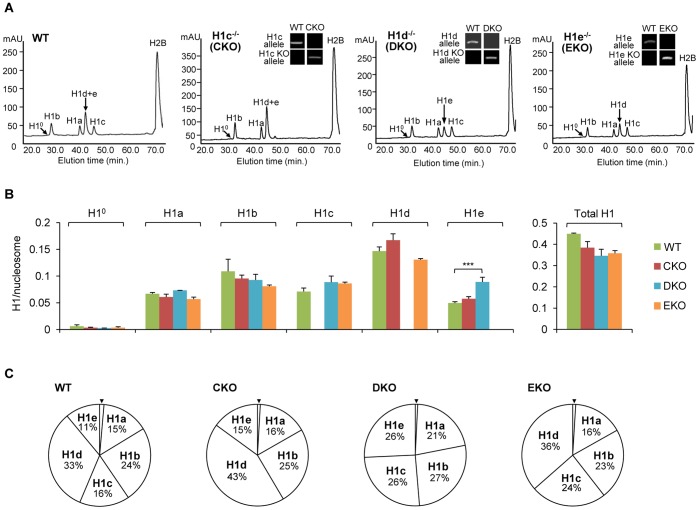
Generation and reverse-phase HPLC analysis of single-H1 KO ESCs. (A) RP-HPLC analysis of total histones from WT and the single-H1 KO ESCs. The identity of the histone subtypes is indicated above each peak. mAU, milli-absorbency at 214 nm. Genotype analyses of single-H1 KO ESCs are shown in insets in respective HPLC profiles. (B) The ratios of individual H1 (left) and total H1 (right) to nucleosome for WT and single-H1 KO ESCs. Ratios were determined from the RP-HPLC and mass spectrometry analyses as described in methods. ***: P<0.001 (C) The percentage of each H1 subtype among total H1 histones for WT and single-H1 KO ESCs. % total H1 for H1^0^ (marked with arrowhead) is equal to or less than 1%.

We next analyzed the total H1 levels and composition of H1 subtypes in these single-H1 KO ESCs. HPLC and mass spectrometry analyses of histone extracts from these cells confirmed the lack of the deleted H1 subtype in the respective H1c^−/−^, H1d^−/−^, and H1e^−/−^ ESCs (Figure 3A). As described previously and shown here [30], [31], quantification of the peaks of each H1 subtype and H2B allows calculation of the H1 to nucleosome ratio (H1/nuc). Such analysis showed that, except for H1e in H1d-KO ESCs, the absolute levels of the remaining H1 subtypes were largely unchanged in single-H1 null ESCs (Figure 3B), indicating that there was little increase or compensation in the levels of the remaining H1s for the lost H1. As expected, undifferentiated ESCs express negligible amount of H1^0^ (Figure 3A), an H1 subtype enriched in differentiating and non-dividing cells [32], [33]. Although relative proportions of H1 subtypes were altered by single-H1 deletion (Figure 3C), the total H1/nuc ratios of H1c^−/−^, H1d^−/−^, and H1e^−/−^ ESCs were comparable with respective values of 0.38, 0.35, and 0.35 (Figure 3B). These ratios were about 25% lower than that of WT ESCs (0.45), but about 50% higher than that of H1 TKO ESCs (0.25) [20]. These single-H1 KO ESCs provide ideal cell resources to ascertain if the effects present in H1 TKO ESCs were caused by any one of the lost H1 subtypes or by the marked reduction in total H1 levels in H1 TKO ESCs.

**Figure 4:**
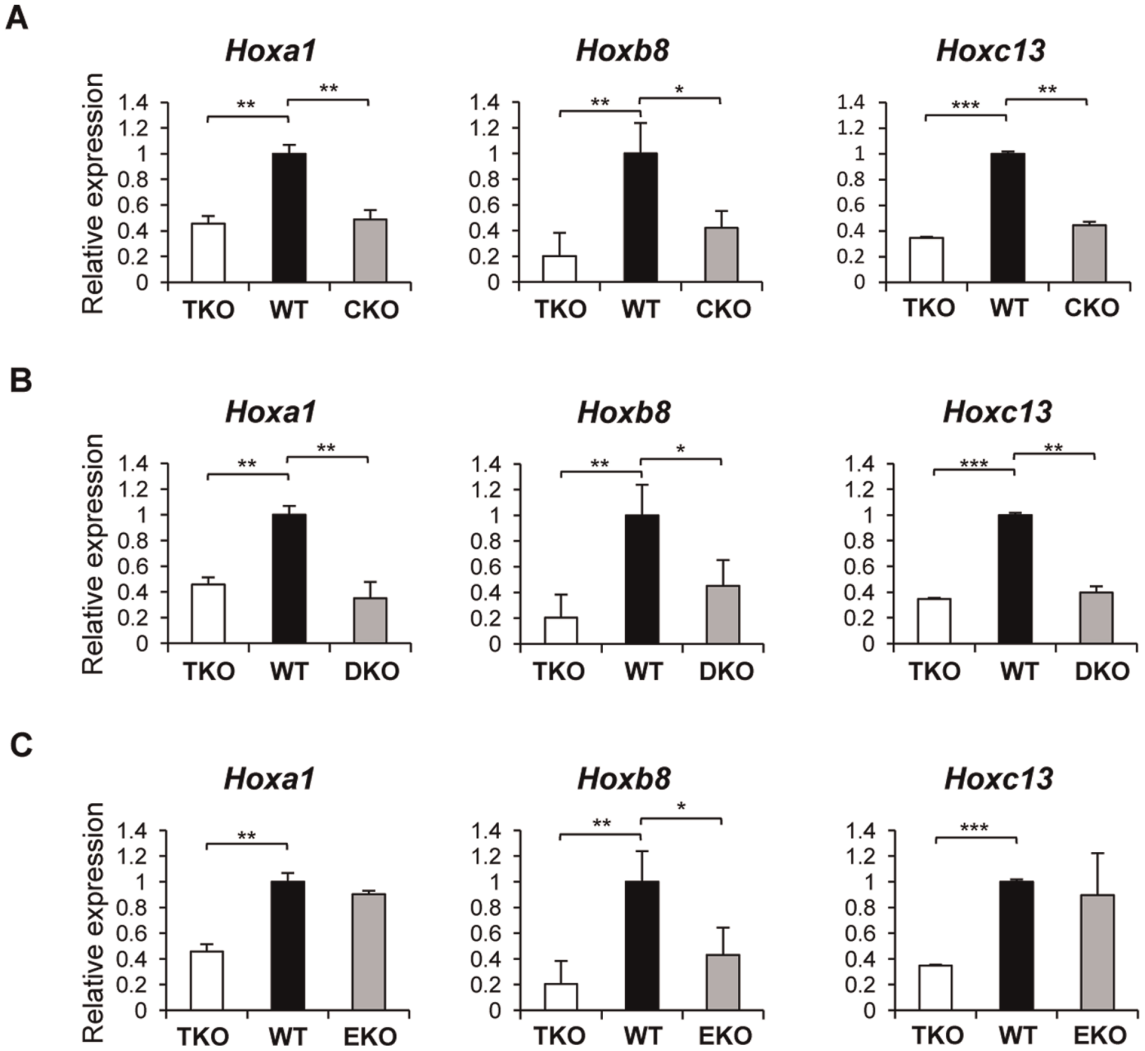
The expression profiles of *Hox* genes in single-H1 KO ESCs. Relative expression of *Hoxa1, Hoxb8, and Hoxc13* in H1c^−/−^ (A), H1d^−/−^ (B), and H1e^−/−^ (C) ESCs were shown. *: P<0.05, **: P<0.01, ***: P<0.001.

We focused our expression analysis in H1 single KO ESCs on the 6 *Hox* genes that displayed reduced expression in H1 TKO ESCs. *Hoxb8* exhibited decreased expression in all three single-H1 KO ESCs, whereas *Hoxa1* and *Hoxc13* had reduced expression in H1c^−/−^ and H1d^−/−^, but not in H1e^−/−^ ESCs compared with WT (Figure 4), indicating that these *Hox* genes are differentially regulated by H1c, H1d and H1e. Interestingly, the expression levels of these *Hox* genes in single-H1 KO ESCs were similar to that in H1 TKO (Figure 4), suggesting that these genes may be especially sensitive to alterations of local chromatin structure or H1 to nucleosome stoichiometry. The other three *Hox* genes did not show consistent expression changes in any of the single-H1 null ESCs, indicating that their expression reduction in H1 TKO ESCs is likely due to the marked reduction of the total H1 levels in TKO cells.

### Dynamic Changes of H3K4me3 and H3K27me3 at Affected *Hox* Genes in H1 TKO ESCs

Trithorax group (TrxG) and polycomb group (PcG) proteins are known to regulate the expression of *Hox* genes [34], [35]. TrxG mediates H3K4 tri-methylation (H3K4me3), corresponding to transcriptional activation [36], [37], whereas PcG directs H3K27 tri-methylation (H3K27me3), correlating with transcriptional repression [22], [38], [39]. In ESCs, many developmental genes display both H3K4me3 and H3K27me3 marks, a “bivalent” chromatin signature for genes poised for expression and important for maintenance of ESC pluripotency [21], [40].

**Figure 5:**
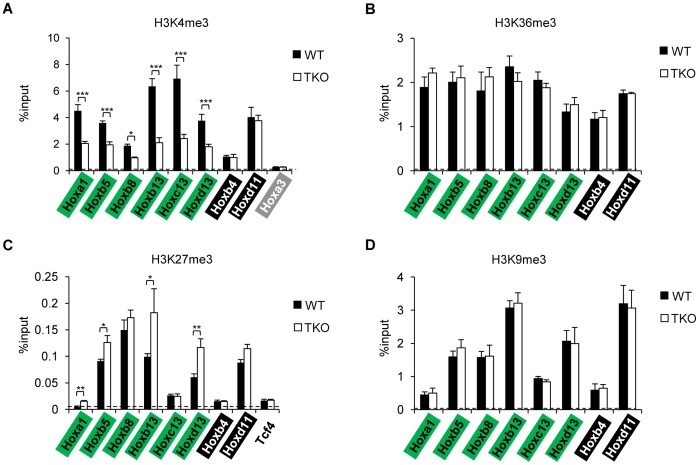
qChIP analysis of histone marks at *Hox* genes in WT and H1 TKO ESCs. The levels of H3K4me3 (A), H3K36me3 (B), H3K27me3 (C), and H3K9me3 (D) were analyzed by qChIP. Promoter regions of the indicated Hox genes were assayed, except for (B), for which gene body regions were analyzed. Dashed lines denote the highest signal level of control IgG qChIP. *: P<0.05, **: P<0.01, ***: P<0.001.

To investigate whether H1 depletion has an impact on bivalent chromatin marks on the 6 *Hox* genes (*Hoxa1, Hoxb5, Hoxb8, Hoxb13, Hoxc13 and Hoxd13*) affected in H1 TKO ESCs, we performed quantitative chromatin immunoprecipitation (qChIP) analysis on the promoter regions of these genes as well as two *Hox* genes (*Hoxb4* and *Hoxd11*) whose expression levels were not altered by triple-H1 deletion. As expected, most *Hox* genes analyzed displayed the bivalent marks in WT ESCs, with higher levels of H3K4me3 and H3K27me3 compared with *Hoxa3* and *Tcf4* (Figure 5A&C), which have been shown to harbor minimum levels of respective histone marks [40]. The levels of H3K4me3 were decreased significantly at all six *Hox* genes affected in H1 TKO ESCs (Figure 5A), but not at *Hoxb4* or *Hoxd11* loci, suggesting that H1 depletion did not lead to a general reduction of H3K4me3 throughout the *Hox* gene clusters. The changes in H3K4me3 level at the promoters of the six *Hox* genes correlated with the reduction of gene expression in H1 TKO ESCs, indicating that the effects of H1 depletion on *Hox* genes may be mediated through regulating the establishment and/or maintenance of specific H3K4me3 patterns. Increased levels of H3K27me3 were observed at 4 of the 6 *Hox* genes affected in H1 TKO ESCs (*Hoxa1, Hoxb5, Hoxb13, and Hoxd13*) (Figure 5C), suggesting that an increase in the H3K27me3 level may also contribute to the reduced expression of these genes. In contrast, H3K36me3, which is enriched at gene bodies of active genes [41], and H3K9me3, which marks heterochromatin and associated with gene repression [42], remained unchanged at all sites after triple H1 depletion (Figure 5B&D), indicating that the effects of marked H1 reduction on H3K4me3 and H3K27me3 (to a less extent) are rather specific. qChIP analysis in single-KO ESCs indicated that H3K4me3 was decreased significantly at the promoters of the *Hox* genes with reduced expression in the respective H1 KO ESCs, but not at unaffected genes, such as *Hoxd11* (Figure S2A). The level of H3K4me3 was not affected by single-H1 deletion at those genes which displayed reduced expression only in H1 TKO ESCs, such as *Hoxb5* (Figure S2A). The increase of H3K27me3 occupancy was more restricted, detected only at *Hoxa1* promoter in H1c- and H1d- KO ESCs with 2–3 fold over WT (Figure S2B). Taken together, our results demonstrate that H1 depletion leads to dynamic changes of the H3K4me3 and H3K27me3 marks, which may regulate *Hox* gene expression.

## Discussion

*Hox* genes encode a large family of transcription factors crucial for body patterning and positioning along the anterior-posterior axis during animal development [1], [43]. Multiple mechanisms have been shown to regulate the spatial and temporal collinearity of *Hox* genes, such as the antagonism between PcG and TrxG proteins [34], [35], local chromatin condensation and reorganization [10], [11], [13], spatial configuration or compartmentalization [14], targeting of miRNAs and long non-coding RNAs (lncRNAs) [44], [45]. Chromatin conformation and compaction appear to be key mediators for regulating the expression of *Hox* gene clusters [10], [11], [13], [14], however, whether changes in chromatin structure have a direct impact on *Hox* gene expression remains undetermined.

In this study, we have taken advantage of a number of mutants, null in one or several major somatic H1 subtypes, with different levels of reduction in total H1 proteins, to investigate the role of H1, a key component in promoting chromatin compaction, in regulating *Hox* gene clusters in mouse embryos and ESCs. We find that depletion of three H1 subtypes leads to the transcriptional reduction of a group of *Hox* genes in embryos and ESCs, and that the reduced expression levels correlate with dynamic changes in H3K4me3 and H3K27me3 marks. This is in contrast to the deletion of PRC1 or PRC2 repressive chromatin complexes, which causes upregulation of specific *Hox* genes in embryos [46]–[48] or ESCs [13], [22], [24].

We first systematically analyzed the impacts of H1 depletion on expression levels of all 39 *Hox* genes in mouse embryos. Consistent with previous findings [9], the posterior genes are not detected by qRT-PCR assays in E8.5 embryos. The 13 affected genes include many paralogous *Hox* gene members (Figure 1B), suggesting a broad effect of H1 on regulation of *Hox* genes. *Hoxa2*, expressed in hindbrain and crucial for trigeminal system development [49], [50], is drastically repressed in H1 TKO embryos. The remaining 12 of the 13 *Hox* genes with reduced expression in H1 TKO embryos are located within paralogous genes *Hox*3*–10*, a region important for axial morphology and patterning [1], [51]–[53]. H1 TKO embryos have significant reduction in total H1 levels and die during midgestation [19]. H1 depletion *in vivo* causes local reductions in chromatin compaction [19], [20]. The finding that all affected *Hox* genes are down-regulated in H1 TKO embryos is surprising because chromatin decompaction and progressive changes in 3D chromatin architecture coincide with activation of *Hox* genes during embryonic development [10]–[14] and thus one may expect that H1 depletion would result in up-regulation of certain *Hox* genes. We believe that the down-regulation of *Hox* genes is a direct effect due to H1 depletion, and contributes to, rather than merely reflects, the growth retardation observed in a fraction of H1 TKO embryos at a later stage [19]. The E8.5 H1 TKO embryos analyzed in this study did not exhibit obvious phenotypic difference compared with WT littermates. It is noteworthy that H1 depletion in embryos did not lead to changes in expression of any of the *Hox* genes on the entire *Hoxb* cluster, which harbors a large intergenic repeat-rich region with a different 3D chromatin structure compared with other *Hox* clusters [14]. Furthermore, similar to our findings from analyzing H1 TKO embryos, H1 depletion in ESCs does not lead to increased expression in any of the *Hox* genes, but causes further reduction in the expression of 6 *Hox* genes. The less prominent effects of H1 depletion on ESCs could be due to the following reasons: 1) ESCs have no or minimum expression of most *Hox* genes; 2) embryos consist of a more heterogeneous cell population which are likely to have very different bulk and/or local chromatin structure at *Hox* gene clusters compared with the undifferentiated ESCs. Indeed, embryos at midgestation have a H1/nuc of 0.74 [19], suggesting a more compact chromatin than ESCs with a H1/nuc of 0.45 [20]; and 3) triple-H1 deletion reduces H1/nuc by 0.34 (from 0.74 to 0.40) in embryos, a larger reduction in total H1 levels than the 0.20 (from 0.45 to 0.25) in ESCs [19], [20].

Importantly, we find that the levels of H3K4me3, a chromatin mark catalyzed by TrxG proteins, are decreased at promoters of all affected *Hox* genes, corresponding to the reduction in gene expression levels of these *Hox* genes in H1 TKO ESCs. Likewise, the correlation of changes in H3K4me3 and *Hox* gene expression extends to the single-H1 KO ESCs, suggesting that individual H1 subtypes may also contribute to epigenetic regulation of H3K4me3 at specific *Hox* genes. The effects of triple-H1 deletion on H3K27me3 are more limited, with mild increase at 4 of the 6 affected genes. We speculate that loss of H1 may lead to changes in occupancy of H3K4me3 methyltransferases/demethylases, and/or affect binding of polycomb complex components to the *Hox* genes [54], resulting in alterations in the histone H3K4 and H3K27 trimethyl marks. It is especially interesting to note that JARID proteins contain an AT-rich interacting domain (Arid) [55], [56] that preferentially binds to AT rich tracts [57] and the matrix attachment region (MAR) [58], a region that is involved in the regulation of *Hox* genes [59] and has a high affinity for H1 binding [60]. However, the levels of JARID1A and JARID1B, two H3K4me2/3 demethylases, do not appear to differ significantly in cellular protein amounts or at affected *Hox* genes in H1 TKO ESCs compared with WT (Cao, Zhang and Fan, unpublished observations). Similarly, H3K4 methyltransferase MLL1 [36] does not display consistent changes by H1 depletion in ESCs (Cao, Zhang and Fan, unpublished observations). Whether any other H3K4me3 methyltransferase(s)/demethylase(s) is responsible for H1 regulated H3K4me3 at *Hox* genes in ESCs remains to be determined. We also cannot exclude additional possible regulatory mechanisms mediated through changes in other epigenetic events upon H1 depletion. For instance, nucleosome positioning is thought to impact DNA accessibility and transcription [61], and H1 depletion leads to a reduction in nucleosome repeat length of bulk chromatin and at specific loci [19], [20]. Nucleosomes are found to be positioned at *Hox* gene clusters, preferentially at 3′ of the expressed *Hox* genes [62], thus the expression of *Hox* genes may be impaired by altered nucleosome positioning in H1 TKO embryos and ESCs. Alternatively, DNA methylation may be affected at *Hox* gene clusters by H1 depletion, which has been shown to affect specific DNA methylation patterns at specific imprinted genes and other loci [20], [63]–[65]. Furthermore, the distance between enhancers or regulatory regions for *Hox* clusters and individual *Hox* genes [66]–[68] may be altered by H1 loss, which in turn reduces *Hox* gene expression.

In order to determine if any of the three deleted H1 subtypes is responsible for the reduction of *Hox* genes identified in H1 TKO ESCs, we derived single-H1 KO ESCs that are null for H1c, or H1d, or H1e. Surprisingly, unlike adult tissues of the single-H1 knockout mice [29], which display no changes in the total H1 levels, single-H1 KO ESCs established in this study exhibit a moderate reduction in the total H1 levels, and a lack of significant compensation for the deleted H1s by the remaining H1 subtypes. Interestingly, the analysis of the 6 *Hox* genes whose expression levels were significantly reduced in H1 TKO ESCs shows that loss of H1c or H1d has similar effects on *Hoxa1*, *Hoxb8*, and *Hoxc13* as triple-H1 deletions. On the other hand, 5 of these 6 *Hox* genes show no expression change in H1e^−/−^ ESCs (Figure 4C). This differential role of the individual H1 subtypes in activating expression of specific genes is reminiscent of the effects of loss of H1a on the expression of non-variegating transgenes in mice [69] and the activation of MMTV promoter by overexpression of H1^0^ and H1c [70]. *Hoxb5, Hoxb13* and *Hoxd13* are not changed in single-H1 null ESCs, suggesting that the expression reduction of these genes in H1 TKO ESCs may be due to additive effects of deficiency of all three H1 subtypes. It is interesting to note that the levels of H3K4me3 are differentially affected at several *Hox* genes, suggesting potential roles of individual H1 subtypes in contributing to the patterns of this histone mark at specific *Hox* genes.

Taken together, the results in this study establish a novel link between histone H1 and *Hox* gene regulation. Furthermore, the reduction of *Hox* gene expression by marked H1 depletion correlates with dynamic patterns of H3K4me3 and H3K27me3 marks. The single-H1 KO ESCs established in this study should be useful cell resources for studying specificity of the individual H1 subtypes in regulating gene expression and epigenetic events.

## Materials and Methods

### Establishment of Mouse Single-H1 KO ESCs and Formation of Embryoid Bodies

Mouse ESCs deficient in histone H1c, or H1d, or H1e were derived from outgrowth of the respective H1c^−/−^, H1d^−/−^, and H1e^−/−^ blastocysts (E3.5) as described previously [20]. Two ESC lines were established for each single KO. Genotyping analysis of WT and KO alleles of H1c, H1d, and H1e loci was carried out as reported [19]. Animal breeding and experimental procedures were approved by Georgia Tech Institutional Animal Care and Use Committee. Embryoid bodies were formed by seeding 1×10^6^ ESCs in a 10-cm ultra-low attachment culture dish (Corning) and cultured for 10 days in media containing Dulbecco’s modified Eagle’s medium (DMEM) (Life Technologies) with 15% fetal bovine serum (Gemini), 0.1 mM MEM Non-essential amino acids (Life Technologies), 55 µM 2-mercaptoethanol (Life Technologies) and 100 U/ml penicillin/100 µg/ml streptomycin (Life Technologies).

### RNA Extraction and Quantitative Reverse Transcription PCR (qRT-PCR)

Total RNAs from ESCs were extracted with Trizol reagent (Life Technologies) according to the manufacturers’ instructions. Total RNAs from embryos were prepared using Allprep DNA/RNA Micro kit (Qiagen). Reverse transcription was carried out using a SuperScript III First-Strand cDNA Synthesis kit (Life Technologies). cDNAs were subsequently analyzed with real-time quantitative PCR (qPCR) using iQ SYBR Green Supermix (Bio-Rad) with a MyIQ Single Color real-time PCR Detection System (Bio-Rad). *Hox* gene specific primers used for qRT-PCR are listed in Table 1.

**Table 1:** Primers for qRT-PCR analysis.

Name	Forward	Reverse
Homeobox A1	tggccacgtataataactcc	aagtggaactccttctccag
Homeobox A2	agtatccctggatgaaggag	aagctgagtgttggtgtacg
Homeobox A3	aacaaatctttccctggatg	cataggtagcggttgaagtg
Homeobox A4	cctggatgaagaagatccac	tctgaaaccagatcttgacc
Homeobox A6	agcagcagtacaaacctgac	agtggaattccttctcaagc
Homeobox A7	tcctacgaccaaaacatcc	aattccttctccagttccag
Homeobox A9	ttgtccctgactgactatgc	aactccttctccagttccag
Homeobox A10	cccttcagaaaacagtaaagc	ttcacttgtctgtccgtgag
Homeobox A11	gacccgagagcagcag	gacgcttctctttgttgatg
Homeobox A13	aaatgtactgccccaaagag	gatatcctcctccgtttgtc
Homeobox B1	acctcctctctgaggacaag	aaatgaaatcccttctccag
Homeobox B2	aagaaatccaccaagaaacc	aagtggaactccttctccag
Homeobox B3	atgaaagagtcgaggcaaac	aagtggaactccttctccag
Homeobox B4	aaagagcccgtcgtctac	ggtagcgattgtagtgaaactc
Homeobox B5	cagatattcccctggatgag	aaccagattttgatctgacg
Homeobox B6	aagagcgtgttcggagag	tgaaattccttctccagctc
Homeobox C6	tcaatcgctcaggattttag	aattccttctccagttccag
Homeobox B8	cagctctttccctggatg	cacttcattctccgattctg
Homeobox B9	taatcaaagagctggctacg	ccctggtgaggtacatattg
Homeobox B13	atgtgttgccaaggtgaac	aacttgttggctgcatactc
Homeobox C4	aagcaacccatagtctaccc	gtcaggtagcggttgtaatg
Homeobox C8	aggacaaggccacttaaatc	tggaaccaaatcttcacttg
Homeobox C9	cgcagctacccggactac	aactccttctccagttccag
Homeobox C10	gtccagacacctcggataac	aatggtcttgctaatctccag
Homeobox C11	aggaggagaacacgaatcc	ttttcacttgtcggtctgtc
Homeobox C12	actccagttcgtccctactc	tgaactcgttgaccagaaac
Homeobox C13	gtcaggtgtactgctccaag	ccttctctagctccttcagc
Homeobox D3	ctacccttggatgaagaagg	aagaggagcaggaagatgag
Homeobox D9	gaaggaggaggagaagcag	tggaaccagattttgacttg
Homeobox D10	gaagtgcaggagaaggaaag	tgaaaccaaatcttgacctg
Homeobox D11	cagtccctgcgccaag	cgagagagttggagtcttttc
Homeobox D12	cttcaaggaagacaccaaag	tgaggttcagcctgttagac
Homeobox D13	gaacagccaggtgtactgtg	gagctgcagtttggtgtaag

### Statistical Analysis

Statistical analyses and P-values were calculated by the Student T two-tailed test. A P-value of less than 0.05 was considered to be statistically significant.

### Preparation and HPLC/MS Analysis of Histones

Total histones were extracted from ES cells as described previously [30], [31]. Briefly, the cells were washed with PBS and harvested. The cell pellet was resuspended in Sucrose Buffer (0.3 M Sucrose, 15 mM NaCl, 10 mM HEPES [pH 7.9], 2 mM EDTA, 0.5 mM PMSF, protease inhibitor) with 0.5% NP-40 and homogenized with a dounce homogenizer (Wheaton). 0.2 N H_2_SO_4_ was used to extract histones from chromatin pellet. HPLC and mass spectrometry analysis of histone proteins were carried out as described previously [30], [31], [65]. Approximately 50 µg histone proteins were injected to a C18 reverse phase column (Vydac) on an Äktapurifier UPC 900 instrument (GE Healthcare). The effluent was monitored at 214 nm (A_214_), and the profiles were recorded and analyzed with UNICORN 5.11 software (GE Healthcare). The values of all peaks were adjusted according to the peptide bonds present in respective proteins. Percentage of total H1 for individual H1 subtypes was determined by the ratio of A_214_ values of individual H1 subtype to that of all H1 peaks. H1 to nucleosome ratio was determined by the ratio of A_214_ values of individual H1 subtype to that of half of the H2B peak.

### Karyotyping

Exponentially growing ESCs were treated with colcemid (Life Technologies) at 37°C for 60 minutes, trypsinized, and harvested. Cells were subsequently resuspended with pre-warmed hypotonic solution (75 mM KCl) and incubated at 37°C for 6 minutes, and fixed as described previously [65]. Fixed cells were concentrated and dropped onto an angled, humidified microscope slide, dried and stained with Hoechst dye for 60 minutes in the dark. Images were collected at a 60x objective on an Olympus Fluorescence Microscope.

### Quantitative Chromatin Immunoprecipitation (qChIP)

qChIP assays were performed as described previously [20] with modifications. The following antibodies were used: anti-H3K4me3 (Millipore 07–473), anti-H3K9me3 (Abcam 8898), anti-H3K27me3 (Millipore 07–449), anti-H3K36me3 (abcam 9050), anti-JARID1A (abcam 65769), anti-JARID1B (abcam 50958), anti-MLL1 (Bethyl Lab A300–086A) and rabbit IgG (Millipore 12–370). Briefly, crosslinked chromatin was sheared by sonication. Pre-blocked Protein G Dynabeads (Life Technologies) were incubated with the antibody and 40 µg of soluble chromatin overnight in 4°C, and subsequently washed with Washing Buffer (50 mM HEPES pH 7.6, 1 mM EDTA pH 8.0, 500 mM LiCl, 0.7% Sodium Deoxycholate, 1% NP-40). Immunoprecipated protein-DNA complexes were eluted and reverse-crosslinked at 65°C, and DNA was purified with a Qiagen DNA Isolation column (Qiagen). The amount of each specific immunoprecipitated DNA fragment was determined by real-time PCR. All samples were analyzed in triplicate in two independent experiments. The percentage of input was calculated by dividing the amount of each specific DNA fragment in the immunoprecipitates by the amount of DNA present in input DNA. qChIP primers are listed in Table 2.

**Table 2:** Primers for qChIP analysis.

Name	Forward	Reverse
Homeobox A1	gggaatccaacagacaccac	tcctcccagtcaatcctctg
Homeobox A1–2	ggcaccctacaccactcact	gaaaccctcccaaaacaggt
Homeobox A3	aattacctccctgcatctcaaa	ttatcagagcagacccacaatg
Homeobox B4	atttccttatccgggaatcg	gtttccgaaagccctcctac
Homeobox B4–2	gtgggcaattcccagaaa	gctggaagccgctctctc
Homeobox B5	taacgaccacgatccacaaa	agagctgccactgccataat
Homeobox B5 –2	cctccaaaatcacccaaatg	gctgagatccatcccattgt
Homeobox B8	gctccgttccaaacacctac	cctccttcaaaggaagcaaa
Homeobox B8 –2	taagcaaggactccctcgtc	gaattacggcgtgaataggc
Homeobox B13	ccctctctttttccaccaca	ttgcgcctcttgtccttagt
Homeobox B13 –2	gagggggtcggaatctagtc	cgcctccaaagtagccataa
Homeobox C13	agctggagcagatcatgtca	gcgctgtcctcatagacgta
Homeobox C13 –2	tgctgaccctgctcactgta	aattctgagcttccctccag
Homeobox D11	tgaacgactttgacgagtgc	ggttggaggagtaggggaaa
Homeobox D11 –2	cctagctcagtggccagagt	agcatccgagagagttggag
Homeobox D13	agctcgaggagccaaagag	gacccaggagttgactttgc
Homeobox D13 –2	gaaaagggtgccttacacca	tgtccttcacccttcgattc
Tcf4	cggatgtgaatggattacaatg	attgttcttcggtcttgttggt

## Supporting Information

Figure S1 **Characterization of the single-H1 KO ESCs and EBs.** (A, B) Karyotypes (A) and phase images (B) of the single-H1 KO ESCs. Scale bar: 50 µm. (C) Characterization of EBs. (i) hematoxylin and eosin staining images of single-H1 KO EBs. Scale bar: 50 µm. (ii) Western blotting analysis of OCT4 in single-H1 KO ESCs and EBs. GAPDH expression levels indicate equal loading of cell lysates. (iii) qRT-PCR analysis of differentiation markers in single-H1 KO ESCs and EBs.
(TIF)

Figure S2 **qChIP analysis of H3K4me3 in single -H1 KO ESCs.** qChIP signals of H3K4me3 (A) and H3K27me3 (B) at indicated Hox genes in single-H1 KO ESCs were normalized to input controls and represented as fold changes over that of WT ESCs. *: P<0.05, **: P<0.01.
(TIF)
