# Supplementary figures and images for: Reduction of Hox Gene Expression by Histone H1 Depletion
Source: PLoS One. 2012 Jun 11;7(6):e38829. doi: 10.1371/journal.pone.0038829 (PMC3372500; doi:10.1371/journal.pone.0038829)

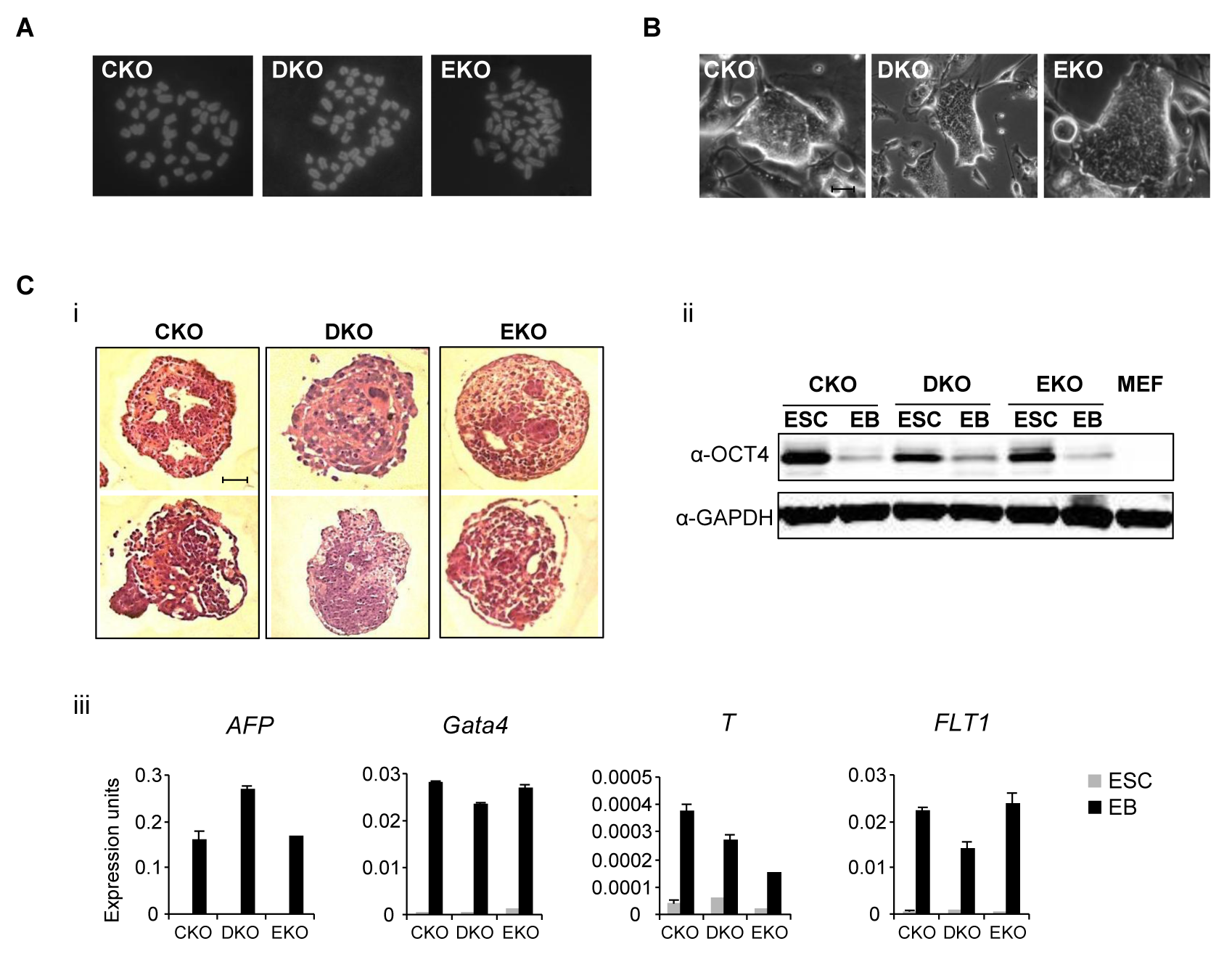

Supplement: Figure S1 — Characterization of the single-H1 KO ESCs and EBs. (A, B) Karyotypes (A) and phase images (B) of the single-H1 KO ESCs. Scale bar: 50 µm. (C) Characterization of EBs. (i) hematoxylin and eosin staining images of single-H1 KO EBs. Scale bar: 50 µm. (ii) Western blotting analysis of OCT4 in single-H1 KO ESCs and EBs. GAPDH expression levels indicate equal loading of cell lysates. (iii) qRT-PCR analysis of differentiation markers in single-H1 KO ESCs and EBs. (TIF) [file pone.0038829.s001.tif]

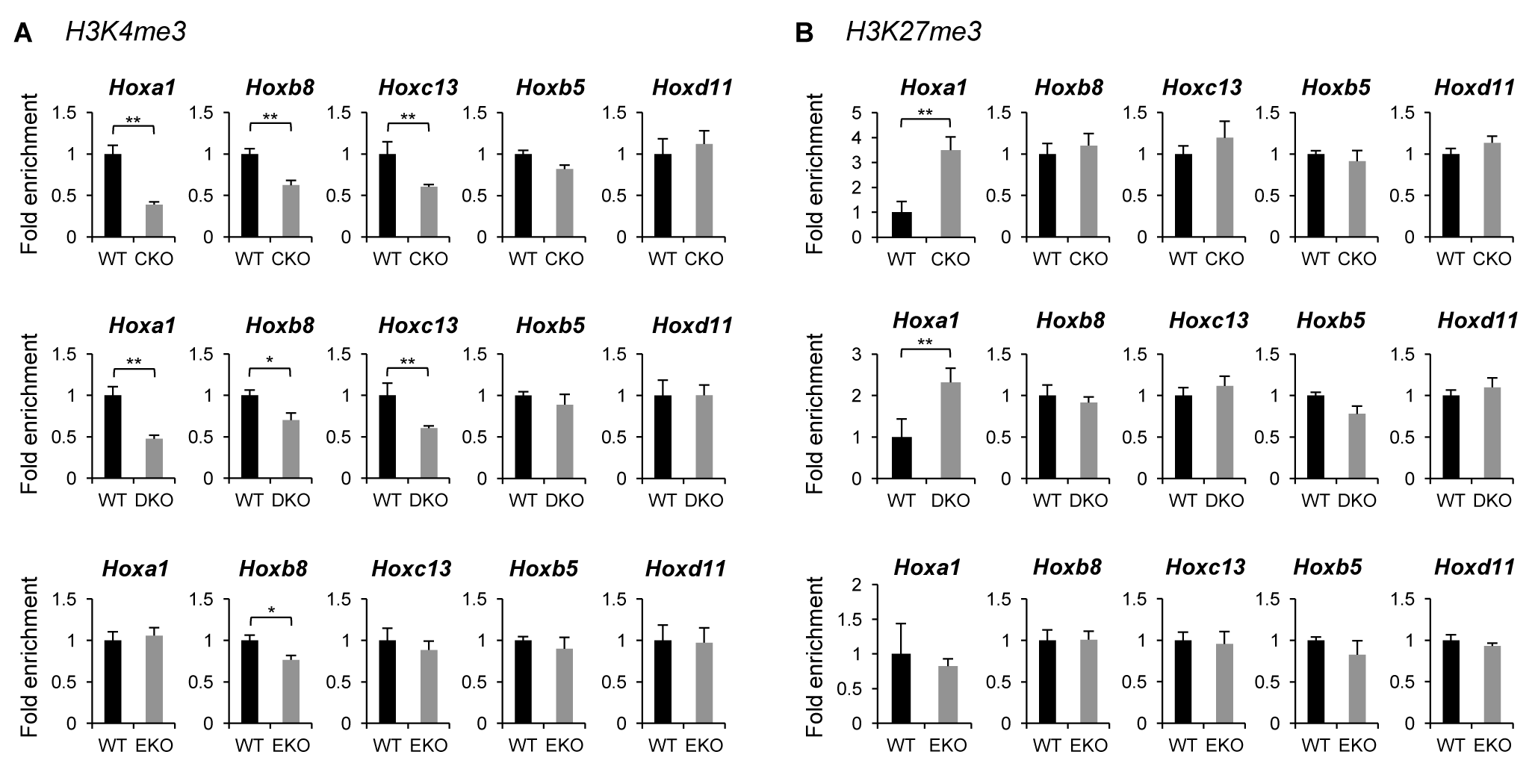

Supplement: Figure S2 — qChIP analysis of H3K4me3 in single -H1 KO ESCs. qChIP signals of H3K4me3 (A) and H3K27me3 (B) at indicated Hox genes in single-H1 KO ESCs were normalized to input controls and represented as fold changes over that of WT ESCs. *: P<0.05, **: P<0.01. (TIF) [file pone.0038829.s002.tif]
